# Systematic anatomical validation of the endoscopic mononostril transethmoid-paraseptal approach to the central skull base

**DOI:** 10.1016/j.bas.2026.106092

**Published:** 2026-05-15

**Authors:** Márton Eördögh, Martin Weidemeier, Gábor Baksa, Lajos Patonay, Daniel Simmen, Henry W.S. Schroeder, Ehab El Refaee, Werner Hosemann, Robert Reisch, Hans Rudolf Briner

**Affiliations:** a Department of Neurosurgery, University Medicine Greifswald, Greifswald, Germany; b Department of Anatomy, Histology and Embryology, Semmelweis University, Budapest, Hungary; c Center for Otorhinolaryngology, Head and Neck Surgery, Hirslanden Clinic, Zurich, Switzerland; d Department of Neurosurgery, Cairo University, Cairo, Egypt; e Department of Otorhinolaryngology, Head and Neck Surgery, University Medicine Greifswald, Greifswald, Germany; f Endomin Center for Endoscopic and Minimally Invasive Neurosurgery, Hirslanden Clinic, Zurich, Switzerland

**Keywords:** Pituitary surgery, Transnasal neuroendoscopy, Transethmoid-paraseptal approach

## Abstract

**Introduction:**

The endoscopic transsphenoidal approach is the standard technique to remove central skull base lesions; however, binostril modifications often require extensive intranasal dissection resulting in sinonasal morbidity.

**Research question:**

The transethmoid-paraseptal approach (TPA) is a mononostril alternative designed to preserve sinonasal function while maintaining adequate surgical exposure. Its systematic anatomical and radiological validation is lacking.

**Materials and methods:**

The TPA was performed on 19 cadaveric specimens to identify anatomical landmarks and assess reproducibility. Based on the dissections, a surgical checklist was developed. Cone-beam CT scans of 43 patients and 9 further cadaveric heads (total: 104 sides) were analyzed using the 3D-Slicer software to identify anatomical variants influencing the approach.

**Results:**

The TPA corridor was successfully established in all cadaveric cases. Consistent identification of key landmarks—e.g. uncinate process, ethmoid bulla, sphenoid rostrum, and sphenoid sinus—was achieved in 100% of specimens, except for one undeveloped ethmoid bulla. Relevant anatomical variants included: fusion of the ethmoid bulla with the basal lamella of the middle turbinate (62.6%), nasal septal deviation (36.6%), posterosuperior ethmoid (Ónodi-) cell (12.2%), conchal type sphenoid sinus (2.8%), maxillary sinus hypoplasia (0.8%). All variants were detectable on preoperative imaging and did not preclude the approach. A conversion to binostril surgery was not required.

**Discussion and conclusion:**

The TPA is a reproducible, anatomically reliable mononostril corridor to the central skull base. Preoperative CT analysis allows identification of anatomical variants that may influence surgical orientation. Further clinical studies are required to compare the surgical outcomes and different transnasal techniques.

## Highlights

• Binostril transsphenoidal skull base surgery may result in sinonasal morbidity.
• We designed a mononostril alternative: the transethmoid-paraseptal approach (TPA).
• It preserves sinonasal function and maintains adequate surgical exposure.
• We performed a systematic anatomical and CT-radiological validation.
• The TPA is a safe, reliable mononostril alternative of the transsphenoidal approach.

## 1 Introduction

The endoscopic transsphenoidal approach is the standard technique for accessing midline skull base lesions (Jankowski et al., 1992). Lesions with lateral localization (e.g., within the cavernous sinus) as well as those demonstrating extensive expansion (e.g., invasive pituitary macroadenomas and large skull base tumors) require a wide surgical exposure. This is best achieved using binostril techniques with bilateral manipulation of the middle and superior turbinates, posterior septectomy, and, in some cases, sacrifice of sinonasal function (Janjua et al., 2012; Cappabianca et al., 2012; Dehdashti et al., 2012). These maneuvers may contribute to postoperative rhinological morbidity, including sinonasal crusting, scarring, and impaired mucociliary clearance (Rioja et al., 2016; Rice et al., 2003).

Several mononostril modifications have been proposed to minimize endonasal disruption. Among these, the transethmoid-paraseptal approach (TPA) creates a unilateral corridor by dissecting along the ethmoid cells, preserving the turbinates and the olfactory region. While the surgical steps have been previously described (Reisch et al., 2017), a systematic anatomical and radiological validation of the approach remains lacking which discusses the sinonasal variant structures that may influence the surgical exposure.

Therefore, the aim of this study was threefold: *firstly*, to delineate constant anatomical landmarks of the TPA through cadaveric dissections, *secondly*, to identify relevant variant structures on the CT imaging, and *thirdly*, to validate reproducibility of the surgical technique through review of cadaveric cases. By integrating anatomical and radiological perspectives, this study provides a comprehensive framework for the safe application of the TPA.

## 2 Materials and Methods

### 2.1 Cadaver studies

We performed the TPA on nineteen cadavers in the Department of Anatomy, Histology and Embryology, Semmelweis University, Budapest, Hungary. There were 9 fresh and 8 formaldehyde-fixed cadavers, an arterial corrosion cast of a skull and another skull. Prior intervention, the vertebral and carotid arteries of the fresh cadavers were injected with red latex. We used 0° and 30° TREND Minop endoscopes (Aesculap AG, Tuttlingen, Germany) for inspection. The documentation was carried out with digital photography (Canon Inc., Tokyo, Japan) and the Aesculap Eddy Full HD system. The dissections were performed with a focus on the landmark structures, their variations, and the associated surgical challenges. Based on the collected data, we developed an anatomical checklist outlining the surgical steps and their corresponding landmark structures.

### 2.2 Surgical technique

The four-hand-technique dissection is performed by two surgeons, one holding the endoscope, the other performing bimanual dissection. The surgical side is typically selected based on the more spacious nasal cavity; however, additional factors such as the sinonasal anatomy and lesion laterality should also be considered. Central tumors exhibiting lateral extension are managed surgically on the ipsilateral side corresponding to the direction of tumor spread. In selected cases of lesions with purely far lateral localization, a contralateral approach may be considered to achieve a better trajectory of dissection. The endoscope is held superior, using the superior rim of the nostril as a pivot. As the base of the nasal cavity is wider, the instruments are placed bilaterally inferiorly. After removal of the decongesting pledgets, the middle turbinate is gently pushed medially to reveal the middle nasal meatus, the uncinate process and the ethmoid bulla. 5 mm anteriorly from the free margin of the uncinate process, a straight incision is performed on the lateral wall of the nasal cavity, then the inferior-medial part of the uncinate process is removed preserving its superior insertion. Alternatively, a dissection from posterior to anterior can be chosen, beginning at the free edge of the uncinate process. Scissors and through-cutting instruments are preferred to avoid a mucosal tear. The resection of the uncinate process opens the ethmoid infundibulum. The anterior-inferior wall of the ethmoid bulla is penetrated with a blunt elevator, then it is removed with a punch. Dissection of other anterior ethmoid cells is performed with punches, forceps and microscissors to expose the basal lamella of the middle turbinate. The orbital lamina (lamina papyracea) and the ethmoid roof are located and left intact. The partial ethmoidectomy is restricted to the inferior area of the ethmoid cells. The middle turbinate's basal lamella is opened inferior and medial to enter the posterior ethmoid cells and arrive at the anterior wall of the sphenoid sinus. These posterior ethmoid cells are accessed near the midline, starting in focus on their medial and inferior parts and then superiorly and laterally with punches. A clear identification of the basal lamella of the superior turbinate is not always possible as this structure regularly merges with adjacent walls (e.g., with the anterior sphenoid wall).

The sphenopalatine artery's branches are coagulated to reduce bleeding or preserved if it is anticipated that a nasoseptal flap will be needed. A triangular bony prominence of the ethmoid, the palatine and the maxillary bone serves as a useful landmark to locate branches of the artery behind it (Eördögh et al., 2018).

The middle and superior turbinates are gently pushed into the space gained by the ethmoidectomy for the wide midline approach. A nasoseptal flap can be prepared by performing a superior and anterior incision on the nasal septum, if this is planned for skull base reconstruction (Rivera‐Serrano et al., 2011). Otherwise, a sagittal 1 cm long incision is performed on the posterior nasal septum at the level of the floor of the sphenoid sinus. The mucoperiosteum is elevated to expose the osteocartilagenous nasal septum, the sphenovomerine suture and anterior-inferior sphenoid wall. Having made an incision through the septum, the contralateral mucoperiosteum is also carefully elevated 5 mm parasagitally, so that the sphenoid rostrum, the inferior part of the sphenoid crest and parts of the vomer can be removed with a chisel or a drill.

The sphenoid sinus is entered paraseptally, near the midline. The landmarks of the central skull base are identified. The sphenoid septa are removed. Complete resection of the sphenoid mucosa is avoided. Enough space should now be available for dissection and resection of the skull base pathology.

If required, the prepared flap can be finally harvested by completing the inferior incision, and can be rotated into the dissected cavity for reconstruction of the skull base defect. Finally, the middle and superior turbinates are repositioned in their natural position. Nasal packing is usually not necessary.

### 2.3 CT analysis

Non-enhanced Cone Beam CT-scans (CBCT) of 43 anonymized patients (22 females and 21 males, mean age: 50.0 years, range: 24-82 years) performed for non-tumorous dental surgical diagnostics were analyzed to study the landmarks using the “3D Slicer” application (Fedorov et al., 2012). In addition to the cadaveric dissections, CBCT scans were available for nine further cadaveric heads (18 sides) including corrosion casts, which were therefore included in the radiological cohort for anatomical variant analysis. A total of 104 head sides have been analyzed. Based on the data of the radiological and cadaveric analysis, variant anatomical conditions in the region of the defined landmark structures were identified. These variants were categorized according to their prevalence and surgical relevance. A variant was classified as “high relevance” if it lies directly within the surgical corridor and is difficult to identify intraoperatively, and, if unrecognized, may result in significant complications such as trajectory deviation or neurovascular injury.

### 2.4 Compliance with ethical standards

All procedures were in accordance with the ethical standards of the institutional and/or national research committee and with the 1964 Helsinki declaration and its later amendments or comparable ethical standards. For this type of study, a formal consent is not required.

## 3 Results

**Fig. 1:**
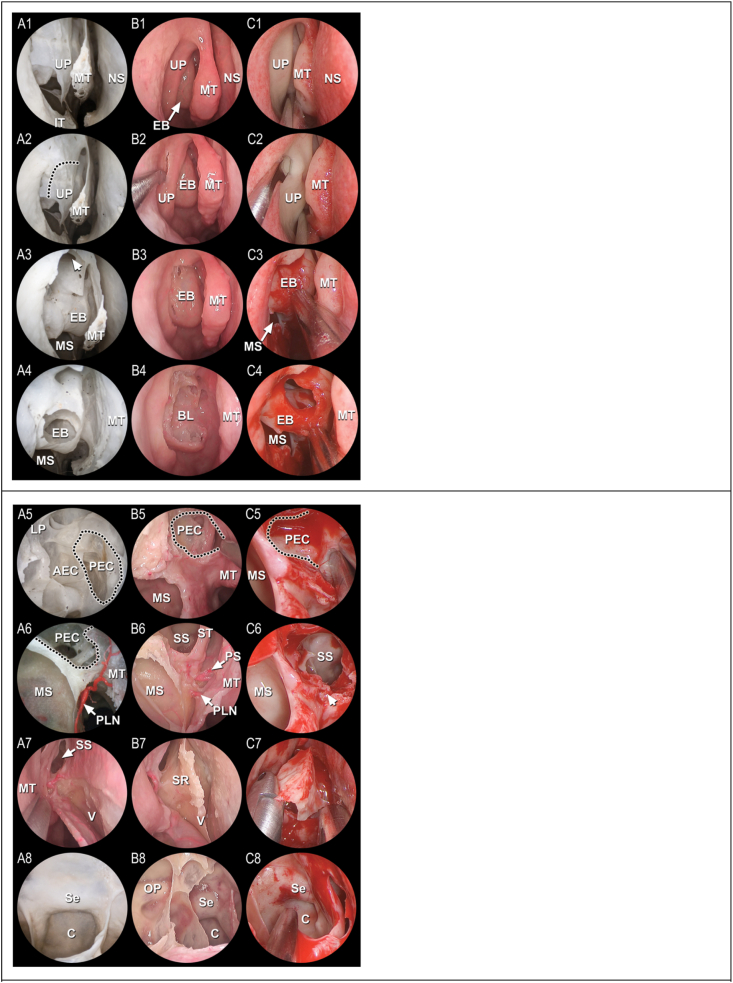
Steps of the transethmoid-paraseptal approach, right side After initial orientation **(A1-B1)**, the middle turbinate is medialized to reveal the middle nasal meatus **(C1)**. The uncinectomy **(A2-C2)** is performed according to the *dotted line*. The middle nasal meatus, the ethmoid infundibulum are revealed **(A3-C3)**; *arrowhead:* ostium of frontal sinus. The bullectomy **(A4-C4)** reveals the middle turbinate's basal lamella **(B4)**, which is opened to enter the posterior ethmoid cells **(A5-C5)***(dotted line)*. The triangular bony prominence (Eördögh et al., 2018) is dissected **(A6-C6)** to explore the sphenopalatine branches *(arrows)*. The sphenoid sinus is reached transethmoidally following the bone superior to the triangle. The middle turbinate is lateralized and the septal mucosa posteriorly incised **(A7)** to expose the sphenoid rostrum **(B7)**, which is removed **(C7)**. The sphenoid sinus is entered **(A8-C8)**. Some images adapted with modifications from another work (Eördögh et al., 2018). A1-5, A8: macerated skull. A6: corrosion cast skull. A7, B1-8: fresh cadaver; C1-8: clinical case as illustration. AEC = anterior ethmoid cell; BL = basal lamella of the middle turbinate; EB = ethmoid bulla; C = clivus; IT = inferior turbinate; MS = maxillary sinus; MT = middle turbinate; NS = nasal septum; OP = prominence of the optic nerve; PEC = posterior ethmoid cell; PLN = posterior lateral nasal branch of the sphenopalatine artery; PS = posterior septal branch of the sphenopalatine artery; Se = sella turcica; SS = sphenoid sinus; SR = sphenoid rostrum; ST = superior turbinate; UP = uncinate process; V = vomer.

**Fig. 2:**
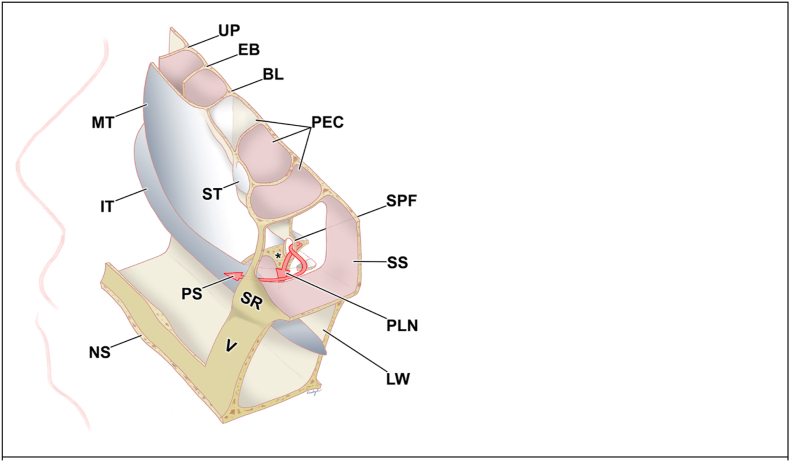
Relevant topographic anatomy of the transethmoid-paraseptal approach, right side View from superior-posterior-medial, based on the model of Stammberger (Stammberger, 2006). Adapted with changes from Eördögh et al. (2021), licensed under Creative Commons Attribution 4.0 International License (https://creativecommons.org/licenses/by/4.0/). BL = basal lamella of the middle turbinate; EB = ethmoid bulla; IT = inferior turbinate; LW = lateral wall of the nasal cavity; MT = middle turbinate; NS = nasal septum; PEC = posterior ethmoid cell; PLN = posterior lateral nasal branch of the sphenopalatine artery; PS = posterior septal branch of the sphenopalatine artery; SPF = sphenopalatine foramen; SS = sphenoid sinus; SR = sphenoid rostrum; ST = superior turbinate; UP = uncinate process; V = vomer; ∗ = triangular bony prominence of the sphenopalatine artery. Some structures are fenestrated (NS, MT, LW, SS) or skeletonized (SPF).

The TPA corridor was established in all 19 cadaveric specimens, demonstrating the anatomical feasibility of this approach in the examined material (Fig. 1). The ethmoidectomy and rostrectomy provided wide access to the sphenoid sinus while preserving the middle and superior turbinates and the entire contralateral nasal cavity. The landmark structures of the TPA showed a high occurrence of 100% with the exception of the ethmoid bulla which was undeveloped in one case (Table 1, Fig. 2).

**Table 1:** Landmark checklist of the transethmoidal-paraseptal approach.

Surgical step	Anatomical structure
Initial intranasal inspection	Nasal septum
	Middle turbinate
Anterior ethmoidectomy	Uncinate process
	Ethmoid infundibulum
	Basal lamella of the middle turbinate
Posterior ethmoidectomy	Superior turbinate
	Anterior wall of sphenoid sinus
Rostrectomy	Sphenoid ostium
	Nasal septum
	Sphenoid rostrum
Sphenoidotomy	Sphenoid sinus

The clinically most relevant variants were the fusion of the ethmoid bulla with the basal lamella of the middle turbinate (62.6%), the nasal septal deviation (36.6%), the posterosuperior ethmoid (Ónodi-) cell (12.2%), the conchal type sphenoid sinus (2.8%), and the maxillary sinus hypoplasia (0.8%), as these may substantially impair orientation and/or pose surgical risks. In contrast, the remaining anatomical variations may complicate the TPA only to a limited extent due to their low prevalence or minor impact on the surgical corridor.

Beyond emphasizing the importance of preoperative CT analysis, Table 2 also outlines specific management strategies for each anatomical variant. None of the identified conditions represented an exclusion criterion for the TPA. Moreover, a conversion to a biportal approach was not required in any case.

**Table 2:** Variant anatomical conditions of the transethmoid-paraseptal approach.

Condition	Prevalence (our material)	Prevalence (literature)	Relevance, potential surgical risk	Management
**Nasal septal deviation**	**T:** 36.6%. **R:** 38.5 % (20 cases). In 55.0% (11 individuals) to the right, in 45.0% (9 cases) to the left side. **C:** 31.6% (6 cases).	22–80% (Vasvári, 2002; Lang, 1988)	**High.** Hinders initial orientation and dissection. In our material, mean mediolateral extension: 8.6 mm (max: 16.3).	Perform contralateral TPA and/or septoplasty.
**A-type uncinate process**	**R:** 58.7% (61 sides). On the right side: 51.9% (27 cases), on the left side: 65.4% (34 cases)∗.	52–54% (Vasvári, 2002; Hosemann et al., 2020)	**Low.** Superior attachment to the lateral nasal wall and orbital lamina. Uncinectomy (if too lateral) may lead to orbitotomy. Irrelevant due to limited medial uncinectomy.	Posterior-to-anterior resection reduces risk. Avoid too lateral dissection.
**B1-type uncinate process**	**R:** 20.2% (21 sides). On the right side: 19.2% (10 cases), on the left side: 21.2% (11 cases)∗.	3–34% (Vasvári, 2002; Hosemann et al., 2020)	**Low.** Superior attachment to the skull base. Uncinectomy (if too superior) might expose the anterior cranial fossa. Irrelevant due to limited medial uncinectomy.	Medial dissection. Avoid too superior dissection.
**Pneumatized uncinate process** (bulla uncinata)	**T:** 5.7%. **R**: 6.7% (7 sides). On the right side: 5.8% (3 cases), on the left side: 7.7% (4 cases). **C:** 0%.	0.4–26% (Vasvári, 2002; Hosemann et al., 2020; Badia et al., 2005)	**Low.** Clearly identifiable. Can narrow surgical corridor. In our material, mean width on the left side: 4.2 mm, on the right side: 4.7 mm.	Avoid lateral penetration.
**Lateral deviation of uncinate process**	0%	Güngör et al. (2016)	**Low.** In contact with orbital lamina. May accompany hypoplastic maxillary sinus. Irrelevant due to limited medial uncinectomy.	No challenge.
**Medial deviation of uncinate process** (duplicated “turbinate” of Kaufmann)	0%	Up to 15% (Vasvári, 2002; Hosemann et al., 2020)	**Low.** Clearly identifiable. Influences orientation during uncinectomy and the identification of the ethmoid bulla.	No challenge.
**Hypoplastic uncinate process**	0%	0.2% (Vasvári, 2002; Güngör et al., 2016)	**Low.** Irrelevant, does not interfere with ethmoid bulla identification.	No challenge.
**Non-pneumatized ethmoid bulla** (torus ethmoidalis)	**T:** 2.4%. **R:** 1.9% (2 sides). On the right side: 1.9% (1 case), on the left side: 1.9% (1 case). **C:** 5.3% (1 case on the right side).	30-38% (Lang, 1988)	**Low.** Resembles the uncinate process.	No challenge.
**Fusion of the ethmoid bulla and the basal lamella of the middle turbinate**	**T:** 62.6%. **R:** 61.5% (64 sides). On the right side: 59.6% (31 cases), on the left side: 63.5% (33 cases). **C:** 68.4% (13 cases)	87% (Eördögh et al., 2021)	**High.** Removal of posterior wall of ethmoid bulla immediately opens posterior ethmoid, earlier as expected. Surgical progress may become faster.	Anticipate early entry into posterior ethmoid. Identify merging and low number of cells on preoperative CT. Keep trajectory inferior-medial.
**Infraorbital** (Haller-) **cell**	**T:** 1.6%. **R**: 1.9% (2 sides). On the right side: 1.9% (1 case), on the left side: 1.9% (1 case). **C:** 0%.	4–46% (Vasvári, 2002; Lang, 1988; Badia et al., 2005)	**Low.** Mimics maxillary sinus opening, increases orbital penetration risk during ethmoidectomy.	Dissection corridor should stay medial.
**Maxillary sinus hypoplasia**	**T:** 0.8%. **R:** 1.0% (1 side). **C:** 0%.	1% (Vasvári, 2002; Papadopoulou et al., 2021)	**High.** Opening of maxillary sinus not recognizable, orbit can be penetrated if dissection is too lateral.	Keep dissection medial.
**Pneumatized middle turbinate**	**T:** 22.8%. **R:** 25.0% (26 sides). Only on the right side: 11.5% (6 individuals), only on the left side: 11.5% (6 individuals). In 7 cases (13.5%): bilateral. **C:** 10.5% (2 cases). 5.3% (1 case) on the right side), 5.3% (1 case) on the left side.	8–53% (Vasvári, 2002; Lang, 1988; Hosemann et al., 2020; Badia et al., 2005)	**Low.** May narrow surgical corridor due to its size.	Partial lateral resection. Preserve medial mucosa when possible.
**Secondary middle turbinate**	**T:** 0.8%. **R:** 1.0% (1 side). On the right side: 1.9% (1 case), on the left side: none. **C:** 0%.	0.8–7% (Thenata and Sensusiati, 2024)	**Low.** Bony protrusion covered by soft tissue in the middle nasal meatus, mimicking the middle turbinate.	No challenge.
**Fusion of the basal lamella of the middle and superior turbinate**	**R:** 14.4% (15 sides). On the right side: 15.4% (8 case), on the left side: 13.5% (7 case).∗	16–31% (Eördögh et al., 2025; Kim et al., 2001)	**Low.** Not relevant due to limited ethmoidectomy.	No challenge.
**Pneumatized superior turbinate**	**T:** 13.8%. **R:** 14.4% (15 sides). On the right side: 15.4% (8 cases), on the left side: 13.5% (7 case). **C:** 10.5% (2 cases). 5.3% (1 case) on the right side), 5.3% (1 case) on the left side.	7–35% (Hosemann et al., 2020; Kajiwara et al., 2020; İla et al., 2018; Cobzeanu et al., 2014; Ariyürek et al., 1996)	**Low.** May reduce visualization and narrow surgical corridor due to its size.	Partial lateral resection if needed. Preserve medial mucosa when possible (olfactory region).
**Posterosuperior ethmoid** (Ónodi-) **cell**	**T:** 12.2%. **R:** 12.5% (13 sides). Only on the right side: 17.3% (9 individuals), only on the left side: none. In 2 cases (3.8%): bilateral. **C:** 10.5% (2 cases). 5.3% (1 case) on the right side, 5.3% (1 case) on the left side.	3–51% (Lang, 1988; DeLano et al., 1996; Weinberger et al., 1996)	**High.** It expands between the anterior cranial fossa and the sphenoid sinus, embodying the optic canal. Dissection of its posterior wall can cause injury of the optic nerve or the internal carotid artery.	Always anticipate. Distinguish from sphenoid sinus on preoperative CT. Avoid posterior wall violation without clear orientation. Consider navigation.
**Inferolateral ethmoid** (Jinfeng-) **cell**	**T:** 1.6%. **R:** 1.9% (2 sides). On the right side: 1.9% (1 case), on the left side: 1.9% (1 case). **C:** 0%.	0.5% (Liu et al., 2019)	**Low.** Not in the surgical corridor.	No challenge.
**Dehiscence or rarefication of orbital lamina**	**R:** exact description on CT not possible. **C:** 0%.	Up to 30% (Hosemann et al., 2020)	**Low.** Risk of orbital injury as its medial bony border is thin or dehiscent.	Keep dissection medial. Avoid lateral pressure.
**Conchal type sphenoid sinus**	**T:** 2.8%. **R:** 1.9% (1 case). **C:** 5.3% (1 case)	1–3% (Lang, 1988, 2001; Hosemann et al., 2020)	**High.** It can hinder the identification of the sella turcica, the prominence of the optic nerve and of the internal carotid artery. Mediolateral and craniocaudal orientation within the sphenoid bone is difficult.	Confirm sellar floor before dural opening. Consider navigation.
**Sphenoethmoidal sinus**	**T:** 3.3%. **R:** 3.8% (4 sides). On the right side: 5.8% (3 cases), on the left side: 1.9% (1 case). **C:** 0%.	1–11% (Eördögh et al., 2025; Rusu et al., 2020)	**Low.** May alter sinus anatomy.	Adjust sphenoidotomy level.

*Total: 71 cases, 123 sides. Radiological cohort: 52 heads, 104 sides. Cadaveric cohort: 19 cases/sides. Abbreviations:****T:****total,****R:****radiological cohort,****C:****cadaveric cohort,****∗:****Cadaveric identification not aimed as only limited ethmoidectomy is performed.*

## 4 Discussion

The endoscopic transsphenoidal technique is the standard transnasal neurosurgical procedure (Jankowski et al., 1992). Its modifications and extensions enhance the exposure of the skull base (Kassam et al.; Kassam et al., 2008). Extensive intranasal dissection provides optimal visibility and surgical access (Lund et al., 2010). Various techniques are available to address this issue, such as the removal of the middle or superior turbinate (Cappabianca et al., 2012) as well as the resection of the posterior half of the nasal septum (Dehdashti et al., 2012). Furthermore, a transnasal biportal approach can be necessary (with or without the mentioned dissection techniques) to remove large or laterally located lesions.

Even though the neurosurgical corridors are clearly defined, the desirable extent of intranasal dissection is still being disputed. Alterations of the normal anatomy may deteriorate sinonasal physiological function. The complications of a voluminous sinonasal dissection are well documented: adhesions, extensive crusting, rhinosinusitis and hyposmia may reduce quality of life. Consequently, there is a trend to minimize the dissection of the nasal mucosa (specially around the olfactory region) and the turbinates to maintain sinonasal function.

Keeping this in mind, the TPA relies on the principles of sinonasal physiology. Tailored dissection of the midline nasal mucosa, the olfactory cleft and the turbinates can maintain olfaction and physiological function. The partial ethmoidectomy provides the space for the middle and superior turbinates to be temporarily lateralized. It is safe as it is restricted to the medial and lower area of the ethmoid cells, away from the orbit and the anterior cranial fossa. The rostrectomy makes extensive septal resection redundant. As a result, a two-surgeon technique is possible through one nostril, even in the case of large tumors.

Compared with the standard mononostril transsphenoidal approach, the TPA provides increased mediolateral working space for four-hand surgical manipulation. This approach allows the endoscope to be positioned at unusual angles and moved extensively, enabling thorough inspection of the lateral tumor margins and potential invasion of the cavernous sinus. Such maneuvers can be technically challenging in mononostril approaches due to limited mediolateral surgical freedom. Certainly, binostril techniques still offer more working space, what may be adequate in extensive lesions, but they may cause more approach related morbidity (Conrad et al., 2016). In contrast to the biportal approach, the uniportal procedure improves postoperative comfort by helping breathing on the other side which remains intact.

For smaller tumors (such as pituitary microadenomas), a standard mononostril transsphenoidal procedure is sufficient. In our practice, the TPA has been applied in cases of large central skull base tumors – pituitary macroadenomas, clival chordomas, craniopharyngiomas –, in which biportal approaches would otherwise be considered. Tumors with purely far lateral localization can be addressed via a contralateral TPA. However, central pathologies with significant lateral extension may not be adequately treated through this mononostril corridor. For example, in cases of giant pituitary adenomas, the restricted working corridor may reduce visualization and increase operative time compared with binostril approaches (Chibbaro et al., 2021a). Similarly, lesions invading the anterior or middle cranial fossa may require extended endoscopic endonasal or alternative approaches, including transorbital routes, which can provide broader surgical access (Chibbaro et al., 2021b).

While the clinical experience (Eördögh et al., 2017) revealed good rhinological outcome, an anatomical analysis of the TPA with focus on the variant sinonasal conditions was lacking. In our experience over the years, neurosurgeons are reluctant to consider this technique because of limited familiarity with the sinonasal anatomy. Interdisciplinary collaboration with ENT is not always available. Therefore, standardized binostril transsphenoidal techniques are frequently preferred.

In this study, we performed a correlation between the anatomical knowledge obtained through cadaveric dissections and the analysis of CT images. We created an anatomical checklist for the surgeon (Table 1) and identified 20 anatomical conditions (Table 2) that should be anticipated as they may lead to disorientation.

The identification of anatomical variants is particularly relevant for surgical planning. Side selection is an important practical consideration in mononostril approaches. Deviations of the nasal septum are common findings, up to 80% (Vasvári, 2002; Lang, 1988); pronounced forms may favor the contralateral side to ensure sufficient working space or need an additional septoplasty. In addition, lesion laterality influences the choice of approach side to optimize the surgical trajectory. The described attachment types of the uncinate process (A and B1) should be understood; however, in general, an incision from posterior to anterior reduces the risk of penetrating the orbit, as the surgeon gradually develops a sense of the extent of the uncinate process (Vasvári, 2002; Hosemann et al., 2020). This technique is particularly safe as the upper portions of the uncinate process at its attachment are intended to be preserved. Variations of the ethmoid bulla bring no danger as they can be easily identified. After the resection of the ethmoid bulla, the surgeon must be aware of potential mergings within the ethmoid cells. Previous analyses demonstrated a frequent fusion of the ethmoid bulla with the basal lamella of the middle turbinate as well as a merging of the basal lamella of the superior turbinate and the anterior wall of the sphenoid sinus (Eördögh et al., 2021, 2025). Consequently, progression through the ethmoid complex may occur more rapidly than anticipated and thus alter the surgical trajectory. Due to the merging, the basal lamella of the superior turbinate cannot always be clearly distinguished. Nevertheless, the reliable identification of the basal lamella of the middle turbinate and the anterior wall of the sphenoid sinus remains possible and provides sufficient anatomical orientation and surgical safety. Dissection within the medial and inferior portions of the ethmoid cells further reduces the risk of iatrogenic injury to the orbit and the anterior cranial fossa, while facilitating early identification of the sphenoid sinus. Maintaining a medial and inferior trajectory also helps to avoid Jinfeng- (Liu et al., 2020) and Ónodi-cells (Ónodi, 1903). The presence of an Ónodi-cell increases the risk of injury to the optic nerve if its posterior wall is inadvertently violated. Low pneumatization of the sphenoid sinus can challenge surgeons to identify the sella. Here, the use of neuronavigation is advised. Given that sphenoid sinus septation patterns are well documented, we did not address this topic in detail. There are no relevant approach-related vascular peculiarities. The sphenopalatine artery's branches are coagulated to reduce bleeding or preserved if it is anticipated that a nasoseptal flap will be needed. A triangular bony prominence of the ethmoid, the palatine and the maxillary bone serves as a useful landmark to locate branches of the artery behind it (Eördögh et al., 2018). The ethmoidal arteries are remote from the dissection corridor. Based on our present findings, all enlisted anatomical variant structures can be anticipated by careful analysis of preoperative CT imaging. They do not represent a significant obstacle for the surgeon. Consequently, no anatomical exclusion criteria for the TPA was established.

The observations in this manuscript underline the need to scrutinize the CT prior to surgery as well as being familiar with morphological variations. The TPA should be considered by neurosurgeons not only as an approach for pituitary adenomas but also larger pathologies of the central skull base. Compared with standard binostril techniques, the TPA aims to reduce intranasal tissue disruption while still enabling a two-surgeon, four-hand technique through a single nostril. However, the unilateral corridor may limit instrument maneuverability and visualization in some cases. Therefore, binostril approaches may remain preferable for extensive central skull base lesions with significant lateral extension.

## 5 Conclusions

This anatomical study demonstrates the feasibility and reproducibility of the TPA to the sphenoid sinus and the parasellar region. Key anatomical landmarks—including the uncinate process, ethmoid bulla, and superior turbinate—were consistently identifiable, while radiological analysis highlighted relevant anatomical variants. The role of this technique as a mononostril alternative for endoscopic transsphenoidal approaches requires further comparative clinical validation.

## Informed consent statement

Not applicable.

## Author contributions

*Conceptualization*: Eördögh, Baksa, Patonay, Simmen, Reisch, Briner; *Methodology*: Hosemann, Eördögh, Weidemeier, Baksa, Patonay, Simmen, Reisch, Briner, El Refaee, Schroeder; *Formal analysis and investigation, data curation*: Eördögh, Weidemeier, Simmen, Reisch, Briner, Hosemann; *Writing - original draft preparation*: Eördögh, Weidemeier, Reisch, Briner, El Refaee, Schroeder, Hosemann; *Writing - review and editing*: Eördögh, Baksa, Patonay, Simmen, Reisch, Briner, El Refaee, Schroeder, Hosemann; Funding acqu*isition*: (none); *Resources*: Eördögh, Baksa, Patonay, Reisch, Briner, Hosemann; *Supervision*: Schroeder, Hosemann, Baksa, Patonay, Simmen, Reisch, Briner, El Refaee, Eördögh. All authors have read and agreed to the published version of the manuscript.

## Institutional review board statement

For this type of study, a formal consent is not required.

## Data availability statement

Data are available from the corresponding author upon reasonable request.

## Funding

This research received no external funding.

## Conflicts of interest

H.R. Briner, D. Simmen, H.W.S. Schroeder and R. Reisch are consultants to KARL STORZ SE & Co. KG (Tuttlingen, Germany). All authors certify that they have no affiliations with or involvement in any organization or entity with any financial interest (such as honoraria; educational grants; participation in speakers' bureaus; membership, employment, consultancies, stock ownership, or other equity interest; and expert testimony or patent-licensing arrangements), or non-financial interest (such as personal or professional relationships, affiliations, knowledge or beliefs) in the subject matter or materials discussed in this manuscript.

## Figures and Tables

**Table:** 

3D	Three-dimensional
CBCT	Cone-beam computed tomography imaging
TPA	Endoscopic mononostril transethmoid-paraseptal approach to the central skull base

## Abbreviations

The following abbreviations are used in this manuscript:
